# A national, geographic database of CDC-funded HIV prevention services: development challenges and potential applications

**DOI:** 10.1186/1476-072X-4-28

**Published:** 2005-11-08

**Authors:** Carol L Hanchette, Deborah A Gibbs, Aisha Gilliam, Kieran J Fogarty, Mark Bruhn

**Affiliations:** 1 Department of Geography and Geosciences, University of Louisville, Louisville KY, USA; 2 RTI International, Research Triangle Park, NC, USA; 3 Centers for Disease Control and Prevention, Atlanta, GA, USA; 4 Department of Interdisciplinary Health Studies, Western Michigan University, Kalamazoo, MI, USA

## Abstract

**Background:**

From 2000–2002, the Centers for Disease Control and Prevention (CDC) funded a study that was designed to improve the information available to program planners about the geographic distribution of CDC-funded HIV prevention services provided by community-based organizations (CBOs). Program managers at CDC recognized the potential of a geographic information system (GIS) to organize and analyze information about HIV prevention services and they made GIS a critical component of the study design. The primary objective of this study was to construct a national, geographically-referenced database of HIV prevention services provided by CDC-funded CBOs. We designed a survey instrument to collect information about the geographic service areas where CBOs provided HIV prevention services, then collected data from CBOs that received CDC funding for these services during fiscal year 2000. We developed a GIS database to link questionnaire responses with GIS map layers in a manner that would incorporate overlapping geographies, risk populations and prevention services. We collected geographic service area data in two formats: 1) geopolitical boundaries and 2) geographic distance.

**Results:**

The survey response rate was 70.3%, i.e. 1,020 of 1,450 community-based organizations responded. The number of HIV prevention programs administered by each CBO ranged from 1 to 23. The survey provided information about 3,028 prevention programs, including descriptions of intervention types, risk populations, race and ethnicity, CBO location and geographic service area. We incorporated this information into a large GIS database, the HIV Prevention Services Database. The use of geopolitical boundaries provided more accurate results than geographic distance. The use of a reference map with the questionnaire improved completeness, accuracy and precision of service area data.

**Conclusion:**

The survey instrument design and database development procedures that we used for this study successfully met our objective. The development of the HIV Prevention Services Database for CDC is an important step toward the implementation of a spatial decision support system. Due to the costs involved in a nationwide survey such as this, we recommend that future data collection efforts use Web-based survey methodologies that incorporate interactive maps.

## Background

The Centers for Disease Control and Prevention (CDC) is among the nation's leading sources of funding for programs to prevent the spread of human immunodeficiency virus (HIV) and acquired immune deficiency syndrome (AIDS). Yet, until recently, CDC had limited information about the geographic distribution of these programs and the extent to which funded services are accessible to the populations at greatest risk of contracting HIV/AIDS.

From 2000–2002, CDC funded a study that was designed to improve the information available to program planners about the geographic distribution of CDC-funded HIV prevention services provided by community-based organizations (CBOs). Researchers and program managers at CDC recognized the potential of a geographic information system (GIS) to organize and analyze information about HIV prevention services. They made GIS a critical component of the study design.

The primary objective of this study was to construct a national, geographically-referenced database of HIV prevention services provided by CDC-funded community-based organizations. To achieve this objective, we were faced with three challenges:

1. to obtain information about geographic service areas across the entire United States and its territories;

2. to design a survey instrument in a manner that encouraged service providers to give accurate information about service area geography; and

3. to design and maintain a database that could handle overlapping geographies, risk populations and prevention services and still be user-friendly for program managers at CDC.

This paper describes how we addressed each of these challenges to achieve our major objective.

### Geographic analyses and health services research

Geography has been a critical component of health care analysis for many decades. In their classic text on health services research, Joseph and Phillips provided a discussion of health care delivery systems around the world, clearly defined the meanings of concepts such as "access" and "utilization," and presented many spatial methods that have been used to analyze them [1]. The text was written when GIS was in its infancy (although developments in computer cartography had been proceeding for several years), but the authors demonstrated how mapping could be used to gain an understanding of the spatial organization of health care providers, such as physicians, and the spatial hierarchy of hospital facilities. They reported on a wide range of methods for measuring accessibility to health services, many of them borrowed from economic geography. These include the location quotient and coefficient of localization, which provide general measures of regional distribution and inequity. Many of the methods used to assess regional accessibility fall into the "distance decay" category, i.e. they involve measurements of cumulative distances between health services and neighbourhoods or other population units and operate under the assumption that distance is a barrier or deterrent to health-seeking behaviour. Other topics covered were health planning and strategies for locating new hospitals and health services.

Many of these methods, such as the computation of physician-patient ratios and location quotients, were demonstrated in Rickets et al. [2]. Rural-urban classification systems were discussed and health care shortage areas mapped. Most of these methods can be applied with geographic information systems.

A geographic information system is an information management system that contains spatially referenced data. Clarke has referred to GIS as 1) a toolbox, 2) an information system, and 3) an approach to science. As a toolbox, a GIS is a software package that contains a variety of tools and functions for processing, mapping and analyzing spatial data. As an information system, it contains a series of databases with observations about features and other entities with known locations. As an "approach to science" it involves the study of the scientific disciplines, such as geography and cartography that have contributed to the development of GIS technology [3].

As an information system, geography thus is the common denominator for disparate data types. Mapping the location of prevention services in relation to HIV incidence, for example, could graphically demonstrate possible gaps in service availability and suggest priorities for locating new service sites. Because maps make complex data more accessible to both experts and non-experts, they can facilitate discussion about issues of access and service needs.

While GIS data are often viewed as maps, the spatial relationships among objects and features in a GIS make it much more powerful than a mere mapping tool. The main reason for this is that spatial data in a GIS are structured in a manner that maintains topological relationships among features such as points, lines and areas. These relationships include adjacency, containment, and connectivity and they allow GIS users to perform analyses among features in a single map layer or multiple map layers [4]. For example, distance could be computed easily between hospitals in one map layer and census blocks in another map layer, to measure accessibility of population to hospitals using a distance decay function. GIS technology can also support spatial query, analysis and modeling functions. Techniques include buffer zone analysis that can estimate the number of persons living within a specified distance of a resource (such as a test site), or the distribution of HIV prevention services in relation to the number of persons living with AIDS.

GIS technology has lead to the enhancement of existing techniques and development of new methods for analyzing health services. GIS can be used to create health service regions based on spatial data using such methods as Thiessen polygons and flow mapping [2]. Spatial interaction models, mathematical programming and GIS network analyses use street data and distance measurements to model flows among patients and health services and are used to allocate patients to services, route emergency vehicles, or strategically locate new facilities [5]. Many of these processes are iterative and can be run many times to examine a host of different scenarios. Of course, GIS analyses would be rather limited without the widespread availability of digital spatial data and health data with geographic identifiers [6].

GIS is increasingly used in public health and health services research. Since 1994, the National Center for Health Statistics has published its bimonthly electronic report, *Public Health GIS News and Information*. In 1999, the *Journal for Public Health Management and Practice *devoted two issues entirely to GIS applications. In May, 2003, the *Annual Review of Public Health *published five articles that, together, constituted a "minisymposium" on the use of GIS in public health. Two issues of the *Journal of Medical Systems *were devoted to GIS in 2004. GIS has been recognized as an emerging technology in the field of public health informatics [7]. Additionally, new journals, such as the *International Journal of Health Geographics *have emerged to meet the demand for research in medical informatics and geographic analyses of health issues.

The CDC has made a commitment to utilizing new technologies to improve health information [8]. The potential of GIS has also been recognized by the Department of Health and Human Services (DHHS). The Healthy People 2010 Objective 23-3 is to "increase the proportion of all major national, state, and local health data systems that use geocoding to promote nationwide use of geographic information systems (GIS) at all levels" [9].

Several recent studies have involved the use of GIS in health services research [10-12]. Of renown also are the Dartmouth atlases of health care, which use data from health care claims databases to map and analyze geographic aspects of health care in the U.S. [13,14]. Most geographic studies are more localized, however, such as an analysis of the accessibility of HIV services in Toronto neighbourhoods or GIS-based assessment of physician shortages took place in a 9-county area of Illinois [15,10].

One of the most important potential uses of GIS technology in evaluation and planning of health care services is as a spatial decision support system (SDSS). SDSS provide information to planners and program managers that allow them to make important decisions about resource allocation. They require the development of a spatially-enabled database, a database management system, and a set of analytical tools, such as those found with most GIS software, for solving problems [16]. The first step in the development of a spatial decision-support system is to develop a spatially-enabled database.

The result of our work has been the development of such a database – a dynamic, national spatial database of locations and corresponding geographic service areas of CDC-funded CBOs that provide HIV prevention services. We termed this database the HIV Prevention Services Database. This database is maintained in a GIS and has enormous potential to provide information to program managers for decision making.

## Methods

We collected data via a questionnaire that was mailed to all HIV prevention service providers funded by CDC during fiscal year 2000. Service providers included those funded directly by CDC and those funded indirectly through cooperative agreements with state or local health departments. While most HIV prevention service providers were CBOs, in some cases, state and local health departments were respondents, describing services that they provided themselves rather than through contracts with CBOs.

The questionnaire prompted respondents to provide information about the following: 1) descriptions of prevention interventions; 2) descriptions of persons served by the intervention; and 3) the location of service delivery and the geographic area in which persons served live. Particularly problematic was the issue of how to ask questions about geographic service areas. While locations of CBOs themselves generally have mappable addresses, how does one ask questions that can provide accurate information about the delineation of geographic service areas? Should respondents draw service areas on a map (to be digitized later) or should they indicate which standard geographic units (e.g. county, ZIP code) best describe their service area? We discuss these issues later in this section.

We pretested a draft questionnaire with HIV prevention providers in Raleigh and Durham, North Carolina. Following revisions suggested by the pretest, we conducted a pilot test in San Diego with HIV prevention program managers in six CBOs. After completing the questionnaire, the program managers participated in debriefing interviews in which they described how they interpreted questions and chose responses and discussed any difficulties they encountered with the instrument. Other revisions were suggested during an expert panel meeting convened at CDC to discuss the survey instrument, database design issues, and analysis.

To maximize compatibility of survey data with other current and planned CDC data collection efforts, response categories for intervention type and persons served were consistent with those of CDC's *Evaluation Guidance *[17]. Using response options shown in Table 1, the following types of data were collected for each prevention program: 1) intervention type, 2) risk population, 3) race and ethnicity and 4) funding source. Multiple responses were allowed for intervention type, risk population, race and ethnicity of the individuals served. For funding source data, we asked respondents whether prevention programs were funded directly by CDC, indirectly through a state or local health department, or both. Although this information was available from CDC and health department data at the CBO level, it was included on the survey instrument to see whether funding sources for specific prevention programs could be identified when respondents received funding from multiple sources.

**Table 1:** Response categories for interventions, risk populations and race/ethnicity of persons served. Data for the following response categories were collected by the survey. Multiple responses were allowed for all categories

**Intervention Type**	**Risk Populations**	**Race and Ethnicity**
• Individual-level interventions • Group-level interventions • Street and community outreach • Prevention case management • Community-level interventions • Health communications/public information • Counseling, testing, referral, and partner notification	• Men who have sex with men (MSM) • MSM/intravenous drug users (IDU) (and other drug users) • IDU • Heterosexual • Mother with/at risk for HIV • General public	• African American • American Indian or Alaska Native • Asian • Native Hawaiian or Other Pacific Islander • Hispanic or Latino • White • More than one race* • Race unknown

### Service area definitions

Data describing intervention types and persons served, combined with the address of responding CBOs, would by itself yield valuable information about the locations of services being provided with CDC funds for specific populations. However, the intent of this study was to describe service area as well as service location. Geographic service areas can be defined in several ways, each of which has ramifications in terms of data collection issues and analyses:

*1. Patient origin*. The service area is defined by compiling actual addresses for persons served. Although this approach provides very precise data, it also involves concerns about respondent burden, confidentiality, and data quality. Many HIV prevention programs do not collect address information; consequently, this approach was not feasible.

*2. Geographic distance*. The service area is defined by the maximum distance from which persons served come to the service. Distance measures are relatively simple in terms of data collection and management. However, because service areas rarely correspond to circular areas described by distance measures, the resulting data can be of relatively poor quality. In some cases, distance measures are converted to administrative units that fall within the specified distance (e.g. all counties that are entirely or partially within a 50-mile radius).

*3. Geopolitical boundaries*. The service area is defined by naming the states, counties, cities, ZIP codes or other administrative units in which services are provided. These units are familiar to most persons and may already be used by respondents in planning and describing their activities. However, geopolitical units may not correspond to service areas that are defined in terms of neighborhoods, and they are sometimes imprecise, such as when a city boundary spans county lines [18].

Based on discussions among the project team and findings from the pilot test, the study team decided to collect service area data in the form of both geographic distance measures and geopolitical units. We did this because there was no clear precedent as to which method would provide the most useful information, and this would provide us with an opportunity to test both. The questionnaire provided respondents with a cascading set of geopolitical unit responses, from which they could list multiple responses at one or more levels of specificity, i.e., multiple counties or a county with additional cities. This list was, for the most part, geographically hierarchical. If, for example, the respondent checked the box for "entire state" and listed "Missouri," no other geographic area response for Missouri was necessary. Response options for distance included six choices ranging from less than 5 miles to more than 25 miles. Response options for service areas are shown in Table 2.

**Table 2:** Service area response options. Service area data were collected in the form of geopolitical units and distance measures. Geopolitical unit responses were listed in hierarchical order. A customized map accompanied each survey to increase accuracy and completeness of responses.

**Geopolitical Description**
• An entire state or territory, or multiple states or territories (Please list the states served:)
• An entire county or island, or multiple counties or islands, but an area smaller than an entire state or territory (Please list the counties served:)
• An entire city/town or multiple cities/towns, but an area smaller than an entire county (Please list the cities and town served:)
• A ZIP code or multiple ZIP codes, i.e. an area smaller than an entire city/town (Please list the ZIP codes served:)
• Tribal lands (Please list the tribal lands served:)


**Distance Specification**

• < 5 miles (specify)
• 5-10 miles
• 10-15 miles
• 15-20 miles
• 20-25 miles
• > 25 miles (specify)

We defined "service area" as the location of persons actually served. In some of the health services literature, this is referred to as "market area" [19]. This may differ from the target area, for which services were planned. For CDC-funded services, the concept of "service area" provided more useful information. The question was phrased in terms of where persons served live, although we did not ask respondents to consult actual address records when choosing their response. For street and community outreach activities, we instructed respondents to describe the area in which the intervention took place because these activities may be directed at transient populations or persons who congregate in a specific area without necessarily living there.

Our instructions to respondents about service area were to specify it as "the area where the majority (roughly 80%) of people receiving this prevention program live," or, for street and community outreach, "where the majority of activities took place." This wording was intended to avoid responses that were skewed toward large service areas by a small number of service users or activities outside the usual service area. In the pilot test, the study team found that this wording elicited responses that more closely represented actual activities.

### Reference map

Each survey package included a custom-made one-page color reference map created for that CBO and generated by an automated GIS routine. The map showed two views of the area surrounding the CBO's location: one identifying cities, counties, and major roads within a 30-mile radius; the other showing a more detailed view of ZIP codes and towns within a 5-mile radius. In both views, we plotted concentric circles at set distances to provide a spatial frame of reference. We based our decision to include these maps on the San Diego pilot test, in which respondents completed service area questions twice: first without a reference map, and then with it. Using a reference map improved data quality in several ways:

*1. Completeness*. Respondents named more cities served when looking at a map that included names of all cities in the county.

*2. Accuracy*. Estimates of distance from the CBO location were more accurate when respondents consulted a map showing distance in 5-mile increments.

*3. Precision*. Respondents described service areas in terms of specific ZIP codes within the city rather than the entire city when using a map showing ZIP code boundaries.

## Results

Surveys were mailed in July 2000. The initial universe was 1,562 CBOs. A number of CBO records in the database were later identified as duplicates or ineligibles (e.g., a CBO that did not provide HIV prevention services in fiscal year 2000), with a resulting survey population of 1,450 CBOs. With follow-up measures such as postcard reminders and callbacks, the survey had an overall response rate of 70.3 percent. In other words, 1,020 of 1,450 CBOs responded to the survey. The number of HIV prevention programs administered by each of these CBOs ranged from 1 to 23. Of the 1,020 CBOs, 432 reported having only one CDC-funded prevention program, but the majority of responding CBOs had more than one. In all, information about 3,028 prevention programs was provided by the survey. We maintained all survey data, actions, and responses in a Microsoft Access control system that was designed specifically for this project.

**Figure 1:**
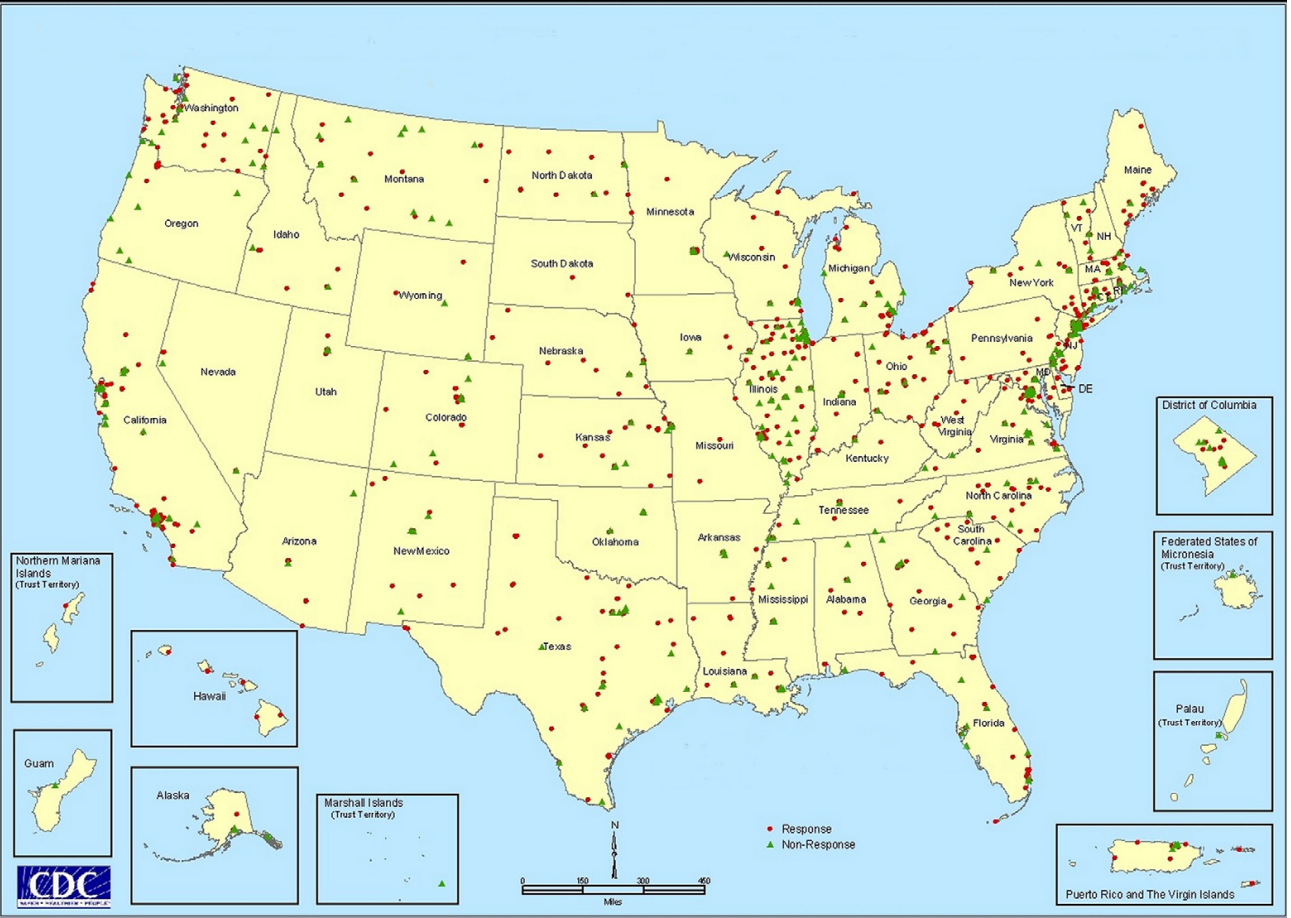
**CBO response to HIV prevention service area survey**. This map shows the location of all CBOs that received CDC funding for HIV prevention services in 2000. CBOs that responded are shown in red; green triangles indicate a non-response.

Figure 1 shows the location of each of the 1450 CBOs in the survey population and their response status. Triangles represent CBOs that did not respond. Particularly notable are the number of non-responses in Illinois and Montana. Montana CBOs were identified late in the data collection process and may not have had enough time to return surveys before that phase of the project ended. In Illinois, the State Health Department acted as an intermediary for the survey and the lack of direct contact for follow-up is likely to have reduced response rates. In this map, non-responses are drawn over responses, which accounts for the pattern present in many of the northeastern cities.

**Figure 2:**
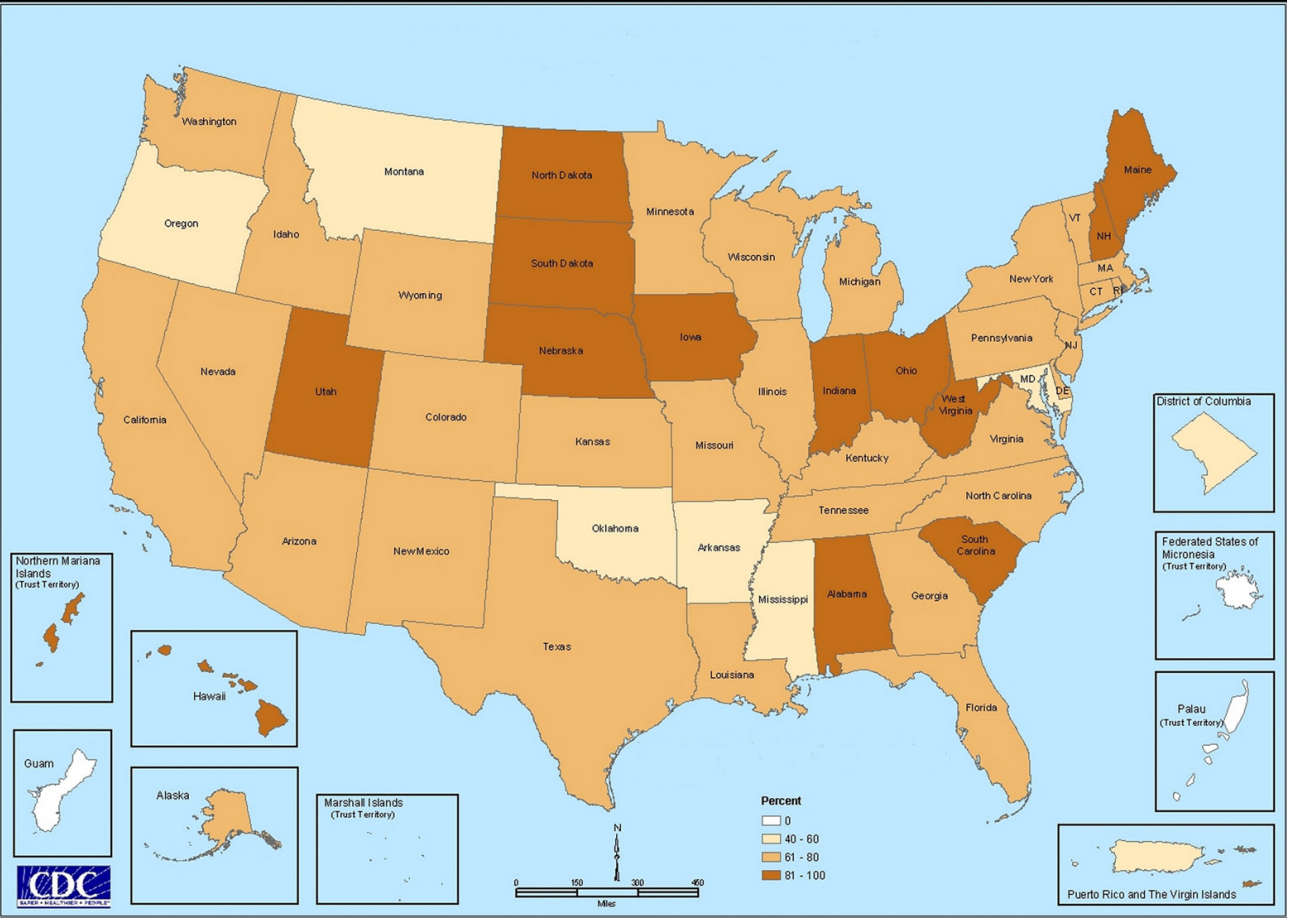
**Response rates by state**. This map shows the CBO response rate, by state and/or territory. Darker shades indicate higher response rates. White indicates no response.

Response rates varied substantially among states, as shown in Figure 2. In the majority of states, 60 to 80 percent of CBOs responded. Higher response rates occurred in some of the Plains states, Utah, the Ohio Valley region, and pockets of the southeastern and northeastern U.S. Eight states/territories had response rates less than or equal to 50%. Response rates are particularly unstable for areas with few CBOs, where responses from just one or two CBOs dramatically influenced the response rate.

We assigned geographic Federal Information Processing Standard (FIPS) codes to service area responses. FIPS codes were developed by federal government agencies to standardize coding for states, counties and other legal and statistical geographic entities. We used the FIPS codes to link the survey data to GIS maps.

Coded surveys were processed by professional data entry staff. Data entry staff wrote a data entry program specifically for this project that included verification, cleaning, and other quality control measures. All data were double-entered and verified. The results of the data entry process were two large text files, one that contained more general CBO information and one that contained all of the HIV prevention program survey responses.

### Database design

**Figure 3:**
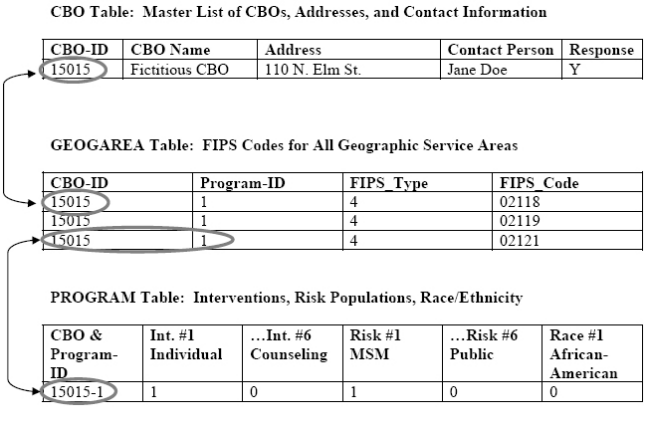
**Database tables and their linkages**. This diagram shows how the three main tables in the HIV Prevention Services Database were linked, or related. CBOs are linked to the GEOGAREA and PROGRAM tables by CBO-ID. The GEOGAREA table contains one record per geographic unit per CBO. This table contains the FIPS codes necessary for linkage to a GIS (map) database.

We converted the text files from the data entry process to a series of 10 Microsoft Access 2000 tables. These 10 tables were developed to normalize the data, (i.e., group them into tables in a formalized procedure to eliminate duplication of information and provide flexibility in table structure for future additions or changes) and to allow linkage to GIS map files via GIS software. Full details of the database design are described in a separate report to CDC and are beyond the scope of this paper [20]. We discuss three important tables in the database, however. These are shown in the Figure 3 schematic, which uses a fictitious CBO. Field names have been changed for readability.

The first table, CBO, contains a master list of CBOs and includes the following information: CBO identifier; name, address and contact person; and information about survey responses. This information was used for survey administration and geocoding.

The FIPS codes for all geographic service area entities (i.e. state, county, city/town, ZIP code and/or Indian Reservation) were stored in a second table, GEOGAREA. This table contained four fields: 1) the CBO identifier, 2) the program identifier (many CBOs had multiple programs), 3) the FIPS type (e.g. state, county, city, ZIP, tribal lands) and 4) the actual FIPS codes. Each geographic unit that represented a portion or all of a service area for a particular program was stored as a single record. In the Figure 3 example, the Fictitious CBO was assigned an identifier of 15015. The service area of this CBO's Program #1 covered three zip codes. Data in the FIPS_TYPE field indicate which GIS base map (i.e., state, county, city, ZIP code, or reservation) to link to. For example, a FIPS_TYPE of 4 indicates that the linkage is to the national ZIP code map layer. The values in the FIPS_CODE field are actual ZIP codes, which can be queried and displayed with the GIS.

A third important table, PROGRAM contains all of the non-geographic information for each program, i.e. intervention type, risk populations, race/ethnicity and funding source. This table is linked, via a combination CBO/program identifier, to the geographic tables. This table also stored values provided for the last survey question, in which respondents were asked to indicate the distance within which the majority of people served lived.

### Geospatial data development

We used a suite of Environmental Systems Research Institute, Inc. (ESRI, Redlands, CA) GIS software products for all spatial data processing and analyses, including ArcGIS 8.12, ArcMap and ArcCatalog. However, the final product of this research – a dynamic spatially-enabled database to be used by CDC program managers – was set up for use in ArcView 8.

We integrated the survey data in the Access database with a series of GIS map layers for subsequent mapping and analysis. These included U.S. states and territories, counties, cities and towns, American Indian reservations, and ZIP code area boundaries. These GIS map layers were derived from two sources: 1) generalized U.S. Census Topologically Integrated Geographic Encoding and Referencing (TIGER) 2000 Arc/Info export files, obtained from the U.S. Census web site; and 2) the ESRI Data and Map series, Version 8.1, which came bundled with ESRI software.

We made some enhancements to the original map layers to incorporate all survey responses. The ZIP code area boundary layer includes some small buffer polygons of ZIP code points that were added for this project. A number of the cities and towns that were identified by the survey participants did not exist in the city/town map layer, so we augmented the places map layer with places found in the online U.S. Geological Survey (USGS) Geographic Names Information System(GNIS). Lastly, a special areas layer was created manually from other background data sets for a few areas specified by survey participants that did not match any of the other background layers.

All responses about geographic services areas were matched to one or more of the geographic boundary files described above. A different procedure was used to develop map layers of CBO and program locations. The CBO and PROGRAM tables in the Access database contain addresses for CBOs and their programs. These addresses were used to derive the CBO and program point locations in geographic coordinates (i.e., latitude and longitude), such as those displayed in Figure 1. CBO and program addresses were address matched by a vendor. Response codes were linked to the address matched CBO data, so response status (i.e., whether the CBO responded to survey or not) of each CBO could be queried and mapped.

The GEOGAREA table contains information about all geographic entities that were indicated, by respondents, to be part of a geographic service area. Responses about geographic distance were stored in the PROGRAM table and linked to the map layer of program locations. The ArcView Buffer Wizard was used to buffer each program point by the corresponding distance estimate to create a new map layer showing service areas based on distance.

**Figure 4:**
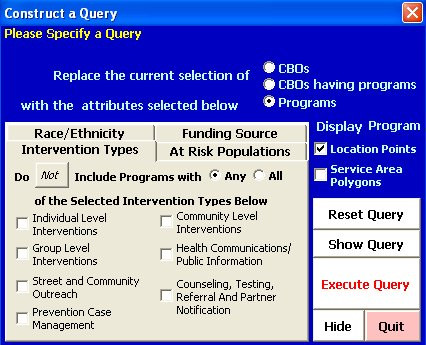
**Query tool interface**. This tool allows users to structure a query based on intervention type, race/ethnicity and risk population, then map corresponding CBO and program locations and/or geographic service areas.

The primary result of this project is the HIV Prevention Services Database, a dynamic, spatially-enabled database that provides CDC with a wealth of information about HIV prevention services that it funds, and a large potential for geographic modeling, analyses, and mapping. This database handles overlapping geographies, risk populations and prevention services. In order to make it user-friendly for CDC program managers, we provided CDC with an ArcView (.mxd) application that automatically loads all of the spatial data (i.e. shapefiles) and tables needed for analysis and mapping. Relationships among tables (i.e. "joins") needed for query and analysis are also maintained in this ArcView application. Due to the wide range of potential database queries, we developed a Visual Basic for Applications (VBA) query tool that makes it easy for users to structure a query based on intervention type, race/ethnicity and risk population. With this tool, the user has the option of mapping CBO and program locations, plus their geographic service areas. The query tool interface is shown in Figure 4.

**Figure 5:**
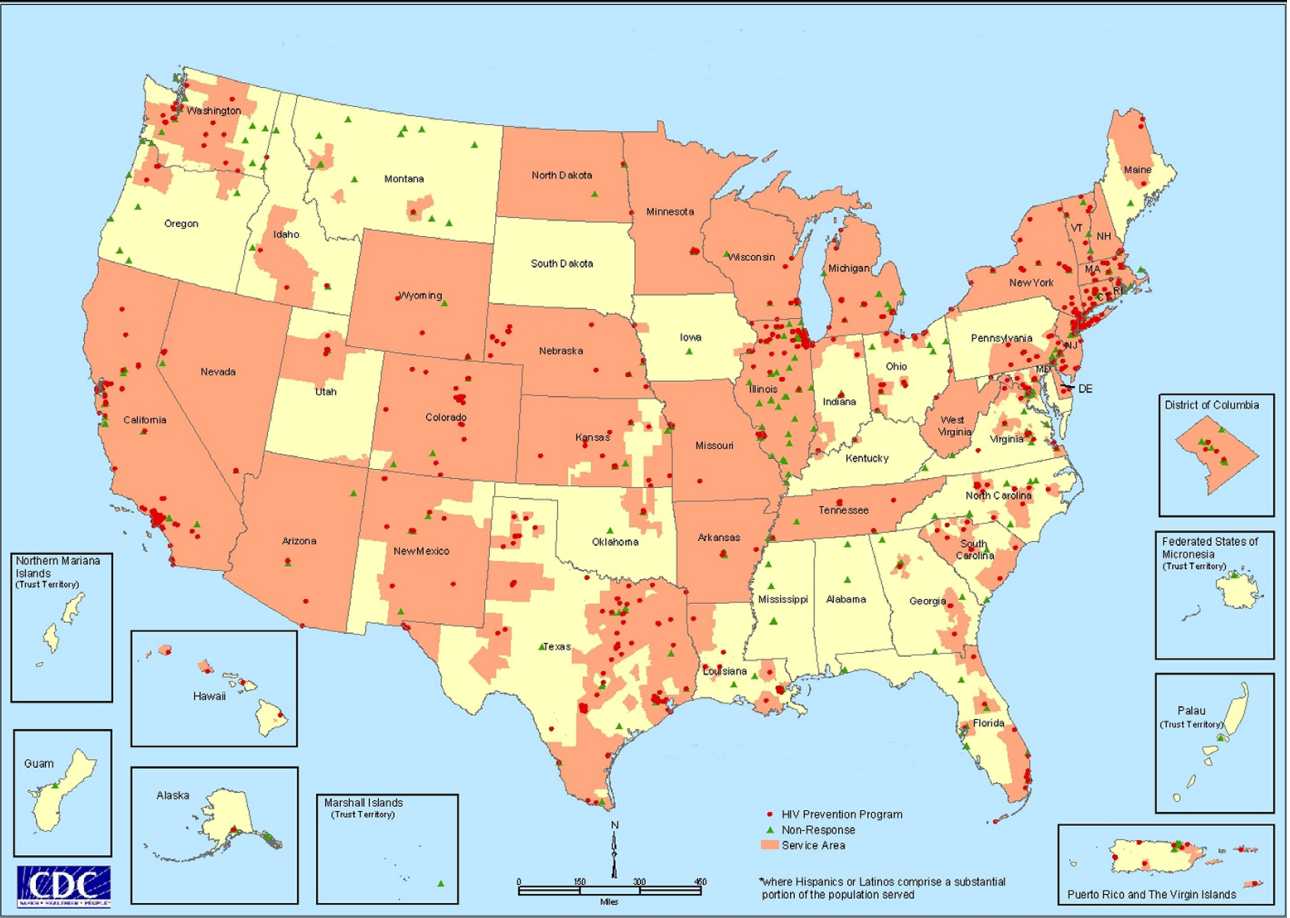
**HIV prevention services to Hispanics or Latinos**. This map is the result of a query to the HIV Prevention Services Database. It shows all areas where HIV prevention services are provided to Hispanics/Latinos. Service areas are drawn in pink. Program locations are represented by red dots. Green triangles represent the locations of CBOs that did not respond to the survey.

The HIV Prevention Services Database presents a wide range of query and display capabilities, based on responses to the survey. For example, Figure 5 shows the geographic service areas of all programs that provide interventions to Hispanic/Latino populations. While the map is national in scale, the zoom and query functions in a GIS allow users to examine geographic areas at any scale. This query was based on program responses to questions about intervention type and populations served.

Queries can be based on geography as well. The question, "Which HIV prevention services are being provided by CDC-funded CBOs in the state of Rhode Island" would produce a map of Rhode Island CBO locations and their service areas and a wide range of information about types of service and risk populations in connected database tables.

The HIV Prevention Services Database is being used by CDC researchers. One analysis has focused on the geographic distribution of services at the national level and another on services to specific populations, such as African Americans.

## Discussion

We successfully developed a national geographic database of CDC-funded HIV prevention services; however, we did encounter several challenges during the data development phase of the project. Some of these were related to data quality and integrity issues. We describe some of these challenges because they are likely to be encountered in other efforts to develop spatially-enabled data.

### Validity of statewide service areas

Many CBOs indicated that they provided prevention services to an entire state. In many cases, this did not seem feasible, and concerns were raised about the integrity of these responses. We developed a set of procedures for confirming the validity of state responses that included 1) using the distance values in the last survey question for validation; 2) a consideration of the size of the state (statewide coverage in Rhode Island is more feasible than that of Texas, for example); 3) examining the type of intervention (e.g. prevention case management vs. health communications;) and 4) telephone callbacks to CBO program administrators by CDC staff.

### Nonexistent geographic entities

In some cases, geographic entities provided by survey respondents simply could not be located in a geospatial database or even an atlas or gazetteer. The most common of these were the ZIP codes. Some CBOs provided ZIP codes that did not exist in the United States Post Office database. Thus, for some CBOs, service area data are missing or incomplete.

### Polygon data not available for some zip codes

Some of the ZIP codes identified by survey respondents did not exist in the ZIP code polygon (area) GIS map layer, but did exist in another GIS layer of points only (i.e., represented by a singlelatitude/longitude coordinate). We made the assumption that these "point only" ZIP codes represented very small ZIP code areas. These ZIP codes were given "area" coverage through the creation of 0.1-mile buffers around their representative points.

### Miscoding of geographic entities by survey data processors

We used a series of GIS queries and logical consistency checks to identify data anomalies. Each time an inconsistency was noted, we examined the original surveys. In a handful of cases, the coders had misinterpreted the respondent's handwriting, and we made corrections.

In spite of some of the challenges we encountered, we successfully developed methods to obtain primary information about CDC-funded HIV prevention services in the U.S. and its territories and were able to develop a dynamic GIS database of CBO locations, service areas and prevention services that is being used by CDC staff to perform analysis and make program decisions. This database, the HIV Prevention Services Database, was delivered to CDC in May 2002.

We need to caution, however, that the response rate to the survey was 70%. While this is a high response rate, we realize that program and service area data are missing for 430 CBOs. Any comprehensive analysis of service provision must take into account the locations of these non-responding CBOs. Additionally, CBO-provided services funded by CDC by no means make up the total of HIV prevention services in the U.S.

The CDC HIV Prevention Services Database was developed to enable CDC researchers to plan and evaluate CBO-provided HIV prevention services. While CDC is using this database primarily to identify gaps and overlaps in service, its potential is broad and includes the following applications:

• geographic analyses of HIV-prevention services to racial and ethnic minorities,

• an examination of HIV prevention services in the context of health disparities as a follow-up to work by Krieger et al. [21],

• use by local health agencies to determine how to integrate CDC-funded services with other community prevention services,

• regional studies of HIV prevention services for areas such as Appalachian Regional Commission counties, and

• analysis of CDC-funding levels vs. assessment of need, based on HIV/AIDS rates and risk populations.

## Conclusion

The development of the HIV Prevention Services Database is a step in the right direction in terms of meeting Healthy People 2010 Objective 23-3 and, ultimately, in the development of a spatial decision support system. We have demonstrated the feasibility of constructing such a valuable, spatially-enabled database nationwide and have successfully transferred the data and technology to CDC for internal use. In terms of defining geographic service areas, we feel that information about geopolitical/administrative units was more useful than distance information although the distance information provided us with a means of conducting logical consistency checks on political/administrative unit responses.

The program data collected for this project were for prevention services provided during fiscal year 2000. We strongly recommend that the HIV Prevention Services Database be updated and maintained on a regular basis. Because of the expense of conducting such a large mail survey, we recommend that future data collection efforts use Web-based survey methodologies that incorporate interactive maps for the delineation of survey areas. These methodologies are being used increasingly in health, social sciences, and educational research [22].

## List of abbreviations

AIDS: acquired immunodeficiency syndrome

CBO: community-based organization

CDC: Centers for Disease Control and Prevention

DHHS: Department of Health and Human Service

FIPS: federal information processing standard

GIS: geographic information system

HIV: human immunodeficiency virus

SDSS: spatial decision support system

VBA: Visual Basic for Applications

## Authors' contributions

CLH and DAG are responsible for the study design. They managed the GIS and survey components of the study, respectively, executed the project, and wrote the final manuscript. This project was the "brain child" of AG. AG and KJF served as CDC Technical Monitors for the duration of the project and directed the study design and implementation. MB developed the GIS database and contributed to the more technical aspects of the final manuscript.

## Notes

1. The HIV Prevention Services Database is in the public domain and is available at no cost for instruction or research purposes.

2. At the time of this writing, ArcGIS 9 was in use.

## References

[B1] Joseph AE, Phillips DR (1984). Accessibility and Utilization: Geographical Perspectives on Health Care.

[B2] Rickets TC, Savitz LA, Gesler WM, Osborne DM, Eds (1994). Geographic Methods for Health Services Research: A Focus on the Rural – Urban Continuum.

[B3] Clarke KC (1999). Getting Started with Geographic Information Systems.

[B4] Vine MF, Degnan D, Hanchette C (1997). Geographic information systems: their use in environmental epidemiologic research. Environ Health Perspect.

[B5] Cromley EK, McLafferty SL (2002). GIS and Public Health.

[B6] Lee CV, Irving JL (1999). Sources of spatial data for community health planning. J Public Health Manag Pract.

[B7] Hanchette CL, O'Carroll PW, Yasnoff WA, Ward ME, Ripp LH, Martin EL (2002). Geographic information systems. Public Health Informatics and Information Systems.

[B8] Centers for Disease Control and Prevention (CDC) Utilizing new technologies to provide credible health information. http://www.cdc.gov/doc.do/id/0900f3ec802454af.

[B9] Department of Health and Human Services (DHHS) (2000). Healthy People 2010: Understanding and Improving Health.

[B10] Luo W (2004). Using a GIS-based floating catchment method to assess areas with shortage of physicians. Health Place.

[B11] McLafferty S, Grady S (2004). Prenatal care need and access: a GIS analysis. J Med Syst.

[B12] Phillips RL, Kinman EL, Schnitzer PG, Lindbloom EJ, Ewigman B (2000). Using geographic information systems to understand health care access. Arch Fam Med.

[B13] Wennberg JE, Cooper MM (Eds.) (1998). The Dartmouth Atlas of Health Care 1998.

[B14] Dartmouth Medical School Center for the Evaluative Clinical Sciences The Dartmouth Atlas Project. http://www.dartmouthatlas.org/current_atlases.php.

[B15] Fulcher C, Kaukinen C (2005). Mapping and visualizing the location of HIV service providers: an exploratory spatial analysis of Toronto neighborhoods. AIDS Care.

[B16] McLafferty SL (2003). GIS and health care. Annu Rev Public Health.

[B17] Centers for Disease Control and Prevention (CDC) (2001). Evaluating CDC-funded Health Department HIV Prevention Programs Guidance Atlanta.

[B18] Simpson K, DesHarnais S, Jacobs A, Menapace A, Rickets TC, Savitz LA, Gesler WM, Osborne DN (1994). Methods for defining medical service areas. Geographic Methods for Health Services Research: A Focus on the Rural – Urban Continuum.

[B19] Rushton G (1999). Methods to evaluate geographic access to health services. J Public Health Manag Pract.

[B20] Centers for Disease Control and Prevention (CDC) (2002). Geoanalysis of HIV Prevention Services Provided by CDC-Funded Community-Based Organizations (CBOs): Final Report DHHS Contract Number 282-98-0022 Atlanta.

[B21] Krieger N, Waterman PD, Chen JT, Soobader MJ, Subramanian SV (2003). Monitoring socioeconomic inequalities in sexually transmitted infections, tuberculosis, and violence: geocoding and choice of area-based socioeconomic measures – the public health disparities geocoding project (US). Public Health Rep.

[B22] McLafferty SL, Clifford NJ, Valentine G (2003). Conducting questionnaire surveys. Key Methods in Geography.

